# A Practical Treatise on the Diagnosis, Pathology, and Treatment of Diseases of the Heart

**Published:** 1860-01

**Authors:** 


### NORTH AMERICAN

### MEDICO-CHIRURGICAL REVIEW.

JANUARY, 1860.

### Analytical and Critical Reviews.

ART. I.—*A Practical Treatise on the Diagnosis, Pathology, and Treatment of Diseases of the Heart.* By AUSTIN FLINT, M.D., Professor of Clinical Medicine, etc., in the New Orleans School of Medicine, Visiting Physician to the New Orleans Charity Hospital, etc. etc. 8vo., pp. 473. Philadelphia: Blanchard and Lea, 1859.

AMONG the names which adorn the medical literature of our country, that of Professor Austin Flint is one of the most prominent. His essays upon a great variety of topics enrich our journals, and his authority in more than one field of research equals that of any living writer. Acute observer, careful recorder, trustworthy reporter, patient thinker and logical reasoner, he is always listened to with respectful attention. It is not easy even for the critical reviewer to speak of his works in any other than the language of eulogy; and we do not now propose so much a critical examination, as a brief and faithful analysis of the volume whose title stands prefixed.

It cannot be said that the diseases of the heart have been of late years neglected or carelessly considered. Some of our most illustrious pathologists and experienced therapeutists have devoted a portion of their time to this class of morbid affections, but no compendious treatise, comprehensive and of easy reference, has been published since Hope's appeared. In the mean while a large amount of material has been accumulating from various sources, to which Professor Flint has contributed his full proportion, and of all which he has in the work before us availed himself with skill and judgment.

The division and arrangement of such a treatise must be in great degree arbitrary, and may generally be left to the discretion of an author of ordinary ability and experience. Professor Flint has chosen to commence with a chapter upon enlargement of the heart. The second describes the lesions of its walls. Valvular lesions and congenital malformations occupy the next three. A chapter is devoted to certain miscellaneous affections connected with structural diseases of the organ. The two following treat of cardiac inflammations. Functional disorder of the heart is the subject of the ninth chapter; and the tenth and last discusses the connected topic of aortic changes and thoracic aneurisms.

Enlargement of the heart includes dilatation and hypertrophy. Hypertrophy may show itself by increase of weight as well as of bulk; and there may be increase in all modes, bulk, weight, and volume. Increase of volume only is ascribed to dilatation, the thickness of the muscular walls being diminished to attenuation. The diagnostic criteria of predominant dilatation are often positive enough; but it is not easy to decide whether the enlargement is or is not accompanied by hypertrophy. Professor Flint decides that the average size of the organ is about four inches in either dimension, being somewhat less in the female than in the male; its average weight being between eight and ten ounces —somewhat less also in the female. The greatest enlargement he has met with reached in weight forty ounces; the vertical and transverse dimensions may be six inches or more. He speaks of fatty enlargement as false hypertrophy; as it is not a hypertrophy at all but a degeneration, we regret his use of the word in that relation. He regards true hypertrophy as an actual "hypergenesis" of the tissue, inasmuch as the size of the muscular fibrillæ is not increased, notwithstanding the augmented bulk or thickness of the muscular wall. If he means that new muscular fibres are superadded, we think the point is not yet settled. In hypertrophy the width is increased more than the length, and the left ventricle oftener affected, and in the greatest degree in most cases, its wall being even two inches thick.

The hypernutrition is the result of undue exercise of the muscular power of the organ, scarcely to be regarded as disease, but rather as a necessary provision for carrying on the circulation under difficulties, and an important conservative provision against over-accumulation of blood in the first instance, as also against the more serious form of enlargement, viz., dilatation. In the great majority of cases the precedent causative conditions are obvious, and exist in the heart itself or the large vessels. When such mechanical causes are not obvious in obstruction or insufficiency of valves, we submit that there is an equally obvious explanation in the repetition of temporary dilatation by such mental emotion or other contingency as excite the heart to transiently undue action; the result of which repeated and vehement efforts is as readily understood as when we have altered mechanism.

There is no difficulty whatever in explaining the production either of uncomplicated hypertrophy or that which is complicated with valvular disorder. Lesion at the aortic orifice, involving contraction and obstruction or inadequateness of the valves and regurgitation, both being often, if not always combined, must occasion undue distention, stimulation, reactive undue effort, and hence hypernutrition. These influences are at first confined to the left ventricle, but soon extend to the right, composed as its wall is in part of the same muscular fibres. Besides this, the left auricle is distended because of the accumulation in the ventricle; it yields and its permanent dilatation gives rise to pulmonary engorgement, thus offering an obstacle to the current through the pulmonary artery from the right ventricle, which is stimulated, dilated, and hypertrophied.

In auricular enlargements dilatation soon predominates, as a rule, over hypertrophy; the same rule ultimately holds good as to the ventricles also, but after indefinite periods.

It is somewhat remarkable that while lesions at the pulmonary orifice occurring after birth are so rare that their occurrence may "almost" be practically disregarded in diagnosis, in fœtal life it is the point of departure for many of the congenital malformations of the heart, the right ventricle being often found enormously hypertrophied.

Not only obstructions at or near the centre of circulation give rise to cardiac enlargement; it may be traced to pulmonary obstruction too, whether from emphysema, chronic pleurisy, collapse or dilated bronchi. Professor Flint doubts, and may well doubt, whether the renal affections found not very unfrequently associated with cardiac disease are, as conjectured, causative of it, or rather consecutive or dependent upon it. We have seen no proofs of their interconnection by causative relation. He gives an interesting instance of their coincidence in a young woman dying after abortion produced in the latter stage of her pregnancy—previous to which she had been well and vigorous. There was here granular degeneracy of the kidney, with uncomplicated cardiac hypertrophy. He also questions, which we do not, the development of hypertrophy by prolonged functional disorder of the heart. He does not deny the tendency, but thinks the increased action is never sufficiently constant or persisting.

He notices, briefly, hypertrophy concentric, centripetal, or with diminution of the capacity of the cavities. He does not think that the marked tonic contraction of the walls accounts clearly for all the appearances, but suggests the weight of the heart as the proper test in such cases.

Of the symptoms of hypertrophy, our author speaks with his characteristic clearness and prudence. The symptomatic phenomena so fluently recited by various writers, he denotes as having been determined inferentially rather than by observed facts.

The physical signs he describes with great neatness as common to both forms of enlargement. 1. Extended and increased dullness in the præcordia on percussion. 2. Altered situation and extent of the apex beat, which is carried to the left of its normal situation and frequently lowered: impulses felt elsewhere than over the apex, as even in the epigastrium; and abnormal force of impulse. 3. Abnormal modifications of the heart sounds, the first sound being notably dull and prolonged as well as exaggerated in intensity—these significant modifications relating more especially to the element of impulsion; in hypertrophy of the right ventricle, an exaggeration of the pulmonary second sound is highly significant; diminished extent and degree of the respiratory murmur and vocal resonance within the præcordia as determined by auscultation, resulting from the displacement or pushing apart of those portions of lung which lie against the sides of the heart in its natural position. 4. Enlargement of the præcordia and abnormal movements, as determined by inspection. 5. Increased size of the chest, as determined by mensuration.

It is obvious that in depending on these two latter signs, we refer to a normal standard, infringed by abnormal projection or prominence of the præcordia or tumor there; but we must remember that very few individuals are truly symmetrical in figure, and that we must allow some margin of irregularity as not interfering with health.

The summary given of the physical signs distinctive of enlargement by *hypertrophy* is clear and concise: 1. Abnormal force of apex beat; impulsion prolonged, sluggish, and strong. In hypertrophy of right ventricle, strong impulse in epigastrium, sometimes communicated to the sternum and extending over the site of the liver. There is a strong heaving movement of the ribs or entire præcordia in hypertrophy, distinguished from the mere shock, more or less violent, due to augmented functional activity (or action) of the ventricles. 2. Exaggeration of the aortic second sound, and of the element of impulsion of the first sound, rendering it in hypertrophy of the left ventricle dull and prolonged as well as intense. In hypertrophy of the right ventricle, exaggerated intensity of the pulmonary second sound; and, in some cases, augmentation of the tricuspid valvular element of the first sound. We collate for comparison, turning over some pages forward, the summary of the physical signs of *dilatation.*

1. The dullness on percussion will show a change of the form of the heart, the tranverse dimensions exceeding the vertical. 2. The apex beat will be found devoid of force, or suppressed; and there will be little or no heaving movement of ribs and præcordia. 3. The element of impulsion of the first sound will be deficient or absent, and the sound short and valvular.

In the treatment of hypertrophy proposed by Prof. Flint, the antecedent pathological conditions which give rise to the morbid affection being immedicable, the indications are laid down as follows, and reasoned out in a manner worthy of his reputation as a wise and experienced practitioner: 1. To prevent or limit the impediments to the circulation dependent on precedent contingencies. 2. To obviate the tendency to dilatation. 3. To quiet undue excitement and irregular cardiac action.

The first indication is to be fulfilled chiefly by negative measures—the avoidance of all excesses in eating, drinking, exercise, mental action; of constipation and alternations of temperature. Blood-letting may be employed, but with great caution; other means of depletion, when that is necessary, are generally preferable, as involving less risk of harm, such as the use of saline laxatives and diuretics, with a somewhat restricted diet. These he regards as free from the evils apt to attend the spoliative effects of losses of blood.

2. Dilatation being the result of weakness of the heart, we must guard against its chief cause—anæmia, or impoverishment of blood. Nutritious diet, exercise within certain limits—we are surprised at the omission of all mention of iron or other tonics here—are the means recommended. 3. Certain remedies are advised to quiet undue excitement and irregular action of the heart, which undoubtedly tend to aggravate all the evils present. We are persuaded of the superiority of the veratrum viride for these purposes to all other remedies. Digitalis, though often useful, is less certain, less regular, less uniform in its effects, and, therefore, much less available. Belladonna and aconite are similarly defective. Prussic acid we have found more worthy of confidence.

Enlargement by dilatation is regarded by our author as the ultimate result of the contingencies which go to produce enlargement in general and hypertrophy. It consists in the yielding of the cardiac walls to the distending force of accumulation of blood; this is determined by functional debility of the organ, or changes in the muscular fibres. Anæmia, the feebleness consequent on pericarditis and adherent pericardium, fatty degeneration and softening, tend to abridge hypertrophy, and favor dilatation.

The symptoms become more definite as this progresses. The apex beat and pulse are feeble and irregular; there is indefinable distress at the præcordia, with dyspnœa and lividity of tongue, lip, and face, and coolness of the extremities. Orthopnœa ensues, aggravated into cardiac asthma attacking in paroxysms; all which are increased by exercise and mental excitement. Cough and expectoration follow; the abdominal viscera undergo passive congestion; the liver is enlarged; granular degeneration of kidneys, attended in some cases with œdema increasing into general dropsy.

With certain qualifications, says Prof. Flint, the indications for the treatment of dilatation are the same as in predominant hypertrophy. Blood-letting is to be employed more rarely, and with greater circumspection—a remark which applies also to other methods of depletion, saline purgatives and duretics, which, perhaps, should be employed to the entire exclusion of venesection. Excessive muscular action, mental and other excitement, must be avoided. External warmth and revulsives to the extremities are called for. Nutritious diet, and, if anæmia be present, iron, reasonable exercise, and avoidance of constipation, are mentioned. So are anodynes, digitalis, and aconite, which, however, must not be pushed beyond the effect of merely tranquilizing the action of the heart, without weakening its muscular power. Paroxysms of dyspnœa are to be palliated by antispasmodics and revulsives, such as the ethers internally, and externally by cupping, sinapisms, and stimulating pediluvia.

Hypertrophy having been thus fully discussed, Prof. Flint goes on in his second chapter to treat of other lesions affecting the walls of the heart. Atrophy, with diminished bulk, is of these the first noticed. The organ is reduced in size and weight occasionally, and the capacity of its cavities diminished. This condition is rare; indeed we are disposed to doubt its existence idiopathically. Our author speaks of it as incidental to chronic diseases of long duration, characterized by gradual progressive emaciation. He does not consider as established its familiarly assumed dependence upon pericardial adhesions and calcification of the coronary arteries. We confess ourselves at a loss to conceive of any grounds upon which it may be regarded, as he intimates, among conservative provisions, and not an evil.

The symptoms are, of course, those which denote feebleness of the circulation, but they are none of them distinctive, being incident also to other lesions, such as dilatation, fatty degeneration, softening, etc. The physical signs, however, are distinctive and diagnostic, consisting in a contraction of the limits of the superficial and deep cardiac regions; in the indistinctness or loss of the apex beat and of the heart sounds, and an extension of the vesicular resonance and murmur over the præcordia. The case is hopeless and immedicable.

Fatty growth and degeneration are the next changes noticed. There may be in health a good deal of fat about the heart, and this deposit does not occasion any inconvenience unless in very large amount; this is most apt to occur after the middle period of life, in persons of what Prof. Gross calls the "adipose diathesis," shown by general corpulence. Beneath the layer of fat the muscular substance may be unchanged, but in general, is pale and softened. Still greater inconvenience arises when the adipose matter is deposited between and among the muscular fibres. Functional debility, atrophy and dilatation, are apt to result. Fatty degeneration differs essentially from mere deposit of adipose vesicles or infiltrated oily matter. The portions of the heart affected assume a yellowish or fawn color, or a mottled aspect in patches, the fat replacing the muscular substance and being deposited in the form of oil globules within the sarcolemma. It thus constitutes, in fact, a form of cardiac atrophy. Examined with the microscope the striæ or transverse markings of the fibres are indistinct or wanting; nay, the muscular substance may disappear, the fibres presenting their outline, but containing fatty matter instead—our author considering the more philosophical explanation of this change to be that which regards it as a process of replacement rather than of conversion. This latter would not be due to perverted nutrition properly, being a chemical, not a vital process. It is doubtful, whether in this replacement, absorption of muscular or deposit of fatty atoms is the first or primary change. Nor is it easy to decide whether the lesion may not depend upon a general diathesis or blood-crasis, as, although it occurs in persons not corpulent, it is usually found associated with similar degeneration in other parts—the liver, spleen, and arterial tunics.

The symptomatic phenomena are not distinctive. The pulse is, of course, feeble; but its slowness—infrequency, we should prefer to say—the number of beats having been reduced to eight or nine per minute, deserves remark. To the other manifestations of cardiac weakness, intermission, irregularity, and, in the advanced stage, great frequency of pulse, general dropsy, oppression, palpitation, and syncope common in other disorders of the organ, our author adds here capillary congestion of the extremities in notable degree. We are pleased to find him skeptical concerning the familiar notion of a fixed pathological connection between attacks of apoplectic and other cerebral disorders and fatty degeneration, or any disease of the heart; or, at any rate, declaring that further clinical observation is necessary to establish it.

Fatty degeneration of the iris, the arcus senilis, he regards with more consideration, as aiding, at least, in the diagnosis here; but speaks of it, with his usual sagacious caution, as a matter for continued inquiry.

The pathological effects of this lesion are also indeterminate. Sudden death may occur in syncope; or it may prove fatal by leading to rupture.

Of this accident, we once saw a remarkable case in a female subject, apparently emaciated in the greatest degree, whose heart was loaded with fat, and considerably softened in its whole structure.

The physical signs may become instructive negatively and by exclusion. There will be no striking increase of volume ; the apex beat will be little or not at all displaced, and more or less enfeebled ; the heart sounds are weak. The concurrence of these signs with the symptoms above noted, will leave little room for doubt, especially if the patient has passed the middle term of life, and has been indolent and luxurious in his habits, or addicted to alcoholic beverages.

In the treatment of fatty growth and degeneration, our objects are threefold. 1. To diminish the effects of cardiac debility. 2. To give tone to the organ. 3. To arrest the accumulation of fat. To fulfill the first indication, cardiac stimulants are required, such as alcohol, ether, and ammonia, which relieve the paroxysms of dyspnœa, syncope, etc., and possibly prevent the apoplectiform coma, which is alleged to attend it. Revulsives are useful. Digitalis and its kindred remedies are of doubtful effect. The second will demand an appropriate system of diet and regimen, and judiciously regulated exercise. Quinia, bitters, and the mineral acids may be employed, and iron, if there be anæmia. Thirdly, the fatty growth—or, to speak more properly, the adipose blood-crasis—may be controlled by the institution of a proper diet, conjoined with habits of exercise. Meat, bread, non-farinaceous vegetables, should be chosen ; oily and sugary substances avoided; and potatoes and rice, as highly farinaceous, taken sparingly. Arrest of progress is all that can be hoped for; the lesion is incurable.

Softening of the heart may be said never to occur idiopathically. It is among the changes liable to take place in the course of fever, especially in typhus and typhoid. It results from inflammation, and can scarcely fail to follow fatty degeneration. In acute or febrile softening, the external muscular layer of the left ventricle has lost all traces of its original structure, and become infiltrated with an adhesive gummy liquid. We know little of its pathological character ; it is non-inflammatory, and does not necessarily lead to serious consequences. It calls for the use of diffusible stimulants.

Induration of the heart is extremely rare, and may be regarded as a mere pathological curiosity. The diagnosis is impossible, and the lesion irremediable. The nature of the change differs in the different examples. Mere condensation, cartilaginous hardening, calcification and ossification are described.

Cardiac aneurism is a pouch-like dilatation, usually seated in the left ventricle, very seldom in the left auricle. It is an extremely rare lesion.

The aneurismal sac, varying in size from that of a small nut to that of the heart itself, consists of layers of condensed fibrin, and contains coagula, and is sometimes studded with calcareous matter. It may be associated with enlargement of the heart, and it is supposed may arise from abscess of the walls of the organ. It may end in sudden death. It does not admit of diagnosis, and is incurable.

Rupture of the heart is perhaps never spontaneous, or the result merely of muscular action. It is incidental to different modes of lesion, most frequently to fatty degeneracy. Its seat is ordinarily the left ventricle. We have alluded to a case in which the opening in a fatty heart was in the right auricle. This was in a female greatly emaciated; but the majority of subjects are males. Prof. Flint met with an instance in which the seat was at the upper part of the right ventricle. The accident is of course fatal, and the diagnosis conjectural.

Carcinomatous and tuberculous deposits are noticed under this head. As unattended by any distinctive symptoms, and very rare, they are considered as of little interest or importance in a practical point of view.

Our author next goes on to treat at length, and in full detail, of the lesions affecting the valves and orifices of the heart. These lesions are interesting and important in many relations, and must be carefully studied. They occur chiefly in the left half of the organ, and are either mitral or aortic; the tricuspid and pulmonic rarely becoming affected after birth.

Aortic lesions generally, but not always, affect each of the three semilunar segments. They may be simply thickened and contracted, or may present on their surfaces vegetations or excrescences varying in size. He once saw fibrinous masses hanging from them downward into the ventricle an inch long. These may be firmly attached, or loose; if washed along with the current of blood, they form the embolia of Virchow and other recent writers. Other morbid growths, cartilaginous and calcareous deposits, may take place at the margin. Occasionally the segments become united at their sides, and, remaining expanded, thus diminish the orifice, as he has seen, to the size of a small crow-quill. These segments may become attenuated and cribriform, and may be ruptured or torn away from their attachments. Mitral lesions are essentially the same; thickening and contraction of the valve, calcareous rigidity, adhesions, vegetations, and excrescences, etc. This valve, however, is subject to aneurisms or circumscribed dilatations, forming pouches protruding into the cavity of the auricle containing coagula or fibrinous laminæ.

The pathological effect of these lesions is threefold: first, obstructing the direct blood-current; second, inducing insufficiency, and thus allowing regurgitation; and third, interfering with the action of the valves by roughening their smooth surfaces. With his usual forethought, Prof. Flint reminds the reader, while observing upon the comparative unimportance directly of the last of these three conditions, that there exist abnormal states, which he must recognize for the physical signs which they give rise to, even if they are not of immediate pathological consequence.

Obstruction and insufficiency may exist separately, but generally concur. Aortic and mitral lesions also are usually met together, but not always. In Prof. Flint's experience, the mitral slightly predominate—a fact which somewhat surprised us. He considers that the changes may be either exudative, organizable and organized, or of fibrinous character, concretion or deposit, probably not organizable. The production of these latter is favored by certain blood-crases, such as the superabundance of the fibrinous element occurring in acute rheumatism. These deposits undergo metamorphosis by the elimination of certain constituents and the addition of others, thus becoming calcareous and rigid, attenuated, perforated, ruptured. Inflammation leads to deposit, both by exudation and coagulation, especially that form connected with acute rheumatism.* But it is not probable that the lesions originate always in inflammation. An exudation similar to arterial atheroma is sometimes the first event; nor is attenuation dependent on inflammation, but on atrophy.

The disturbance of the circulation, the primary effect of these obstructions or regurgitant lesions, gives rise to a train of secondary or remote pathological consequences, manifesting themselves locally in the heart itself; generally in the wide distribution of the vascular system, and spreading to the respiratory, nervous, digestive, and genito-urinary: in which order our author discusses them.

Sooner or later they lead to enlargement of the heart, by inducing over-distention of the cavities and over-excitement of the organ. The ordinary manner in which this result takes place is given. Mitral lesions first dilate the left auricle; next, the right ventricle; next, the right auricle; lastly, the left ventricle. This rule, however, is subject to variations. Thus the left ventricle is sometimes diminished in size when the rest of the organ is enlarged—a fact explained by the diminished supply of blood received when the mitral orifice is greatly obstructed.

* In a valuable note the proportion is thus stated: Of 61 cases of valvular lesions, rheumatism is recorded to have occurred previously in 43. Of 29 cases of mitral lesions, it had occurred in 20. Of 14 cases of aortic lesion, in 7. Of 18 cases of concurrent lesion, both mitral and aortic, in 16.

Nay, the mitral orifice has been reduced to the size of a crow-quill, without notable enlargement of any of the cavities.

Enlargement from aortic lesions always commences at the left ventricle. Obstruction without regurgitation produces hypertrophy; when regurgitation predominates, there will be dilatation.

Concurrent causes modify the result of lesions; thus if there be true pulmonary emphysema, the enlargement of the right will be as great or greater than that of the left ventricle. Nor is the enlargement always proportioned to the obstruction or insufficiency observed—a point much dwelt on by Gairdner, who gives some extraordinary examples in illustration.

In considering the influence of the rare structural changes in the tricuspid and pulmonary valves, Prof. Flint regards it as reasonable to presume, with Hunter, that the difference of arrangement between the mitral and tricuspid valves has some important object; and with Adams and King, that it is not improbable that this object is to permit a certain amount of regurgitation.

Obstruction of the coronary arteries, which he includes among the effects of valvular lesions, he ascribes to the encroachment of masses of fibrinous or calcareous deposit over their mouths, or within these vessels. Enfeebled muscular action and impaired nutrition must be the consequence.

Here also he treats of cardiac pain, palpitation, abnormal pulse, venous turgescence and venous pulsation. Pain, he affirms, is not a prominent symptom of vascular lesions; nay, absence of pain is the rule. Even when endocardiac inflammation is present, no form of pain, tenderness or soreness is always attendant. A sense of constriction, uneasiness or indefinable distress is oftener met with, but must not be thought distinctive of organic disease, being quite as likely to occur in connection with merely functional disorder. Palpitation is not, at least until the more advanced stages of enlargement, a constant symptom; the patient may not notice it, even when it is objectively marked; other things being equal, it is proportioned to the hypertrophy of the left ventricle. In functional disorder it gives great anxiety and alarm; and, therefore, if much complained of, it gives presumption against the existence of organic disease.

The pulse will be weak in general proportion to the degree of regurgitation, and this feebleness will be in contrast with the impulse of the heart felt at the præcordia. Mitral contraction renders the pulse not only weak, but often intermitting, irregular, and unequal. Intermission may be observed when the apex beat is uninterrupted, the wave passing through the contracted opening being too small to be appreciated. Our author repeats the statement found in the books, that there are instances in which the pulse, intermittent in health, ceases to be intermittent in disease attended by febrile movement, the intermission returning with the restoration of health. We will deny no fact, however mysterious and improbable, when observed and asserted by competent authority, and will yield our skepticism before a single case recorded in detail by Professor Flint. But we are not clear that he affirms himself to have been a witness here, and we are not convinced by the vague rehearsal of such strange things; waiting until a definite example has been properly watched and noted.

Among the other modifications of pulse, under these contingencies, the author is disposed to agree with Dr. Henderson, in regarding the prolongation of the interval between the radial pulsation and the heart's impulse, as significant of aortic regurgitation. He met with an instance in which this interval was longer than that between the first and second sounds of the heart; the radial pulse being, in fact, in much closer relation to the diastole than to the systole.

The turgescence of veins, and venous pulsation, though wanting in precise diagnostic significance, possess considerable value when taken in connection with the physical signs which establish the nature and seat of organic lesions. They manifest an influence on the systemic circulation, which involves a liability to other morbid results, dropsy, hemorrhage and extravasations. The diastolic movement of the veins in venous pulsation is due to a retrograde current, impressed by the contraction of the right ventricle. Not uniformly confined to the veins of the neck, it is rarely seen except in the jugulars, and is usually observed on the right side. The cervical veins may, however, pulsate under the contraction of the right auricle also ; an interesting subject, as our author thinks, for further clinical study.

Of the diffused vascular disorders developed as ulterior results of vascular lesions, cardiac dropsy receives the first notice. This serous exudation affects the several cavities with a frequency, stated in order, as follows: the peritoneal, pleural, pericardial, arachnoid, subarachnoid, and the tunica vaginalis. The last of these must be rare. We have never found it associated with general dropsies. The exudation, or exosmose, is the simple consequence of the mechanical pressure on the distended coats of the vessels; thus they are rendered permeable to the serous or watery portion of the blood. The relation between the degree of the valvular lesions, and the dropsies which follow them, is liable to be greatly modified by concurrent conditions in the system. The principal of these is disease of the kidney, renal and cardiac dropsy being thus combined. Anæmia or hydræmia also aids to determine the occurrence of dropsy.

Embolia engages our author's attention for a moment. The detached deposits, migratory plugs, or *emboli* of Virchow have but recently attracted the notice of the profession, and as yet not much is definitely known concerning their pathological history. His extensive reading affords some interesting observations on the subject, which needs, however, further inquiry and elucidation.

The engorgement of the lungs arising from valvular lesions gives rise to dyspnœa, cough, and hæmoptysis, pneumorrhagia and œdema. When the dyspnœa comes on in paroxysms, it constitutes cardiac asthma. It is greatly modified by coincident affections, as emphysema, pleuritic effusions, etc.

Professor Flint does not admit the truth of the common impression that cerebral disorders in general belong to the history of cardiac disease. He affirms on the contrary that they occur in only a small proportion of cases, and that, even if they were more frequently present, they would possess small diagnostic significance.

A certain amount of headache, vertigo, tinnitus, dullness and drowsiness may occur in cases of valvular lesion, on account of abnormal fullness of the cerebral veins. Sleep is frequently imperfect, and disturbed with bad dreams; but this is rather owing to the dyspnœa present than to cerebral disorder. So also of the globus hystericus noticed in some cases.

The mental condition of patients laboring under grave organic disease of the heart is characteristic. They are generally apathetic, free from apprehension, indifferent or incredulous when told of their condition. Toward the close of life there may be some degree of mental aberration, but delirium cannot be reckoned among the events belonging to their natural history.

The digestive and nutritive systems do not escape the pathological influence of the venous congestion dependent upon vascular lesions. Cook of Transylvania, as is well known, ascribed as many diseases to this venous congestion as Broussais to gastro-enteric irritation, or Brown to asthenia. But our author discriminates. He admits that morbid affections of the digestive system do not hold a prominent place here. In a large majority of the histories of one hundred cases analyzed by him, nothing of importance was noted with reference to this system. But it is not always thus. Enlargement of the liver is an effect occasionally of valvular lesions and enlargements of the heart. The augmented size of that great organ is sometimes remarkable, and its variations of bulk at different times. Jaundice may attend, but rarely. Cirrhosis is less frequent than is generally affirmed. The pressure of the portal branches or interlobular veins on the biliary tubes may however give rise to an undue accumulation of bile. Sections of the organ then present that peculiar aspect known as the "nutmeg liver."

So far from diminished nutrition being one of the pathological effects of valvular lesions, they are rather characterized by the absence of notable deterioration in that respect. When their origin dates in early life, and enlargement of the heart takes place before puberty, the body may attain to a full development.

Congestion of the kidneys is generally the result of valvular disease with dilatation of the right heart. The urine is scanty; its solid constituents relatively increased; the lithatic deposits abundant; and albumen is present. Bright's disease is sometimes associated with this condition, though less frequently than is supposed. Indeed the two affections are so rarely united that it may be doubted whether there is established any pathological connection between them.

Of the countenance and external appearance of the body in valvular disease, Prof. Flint remarks that a dusky hue of the face, combined with œdema, is quite distinctive of cardiac as contrasted with renal dropsy, altering the visage and its expression so much that the person becomes scarcely recognizable, and resembles a cadaver after strangulation. The urgent dyspnœa induces the expression of great anxiety and distress. But anæmia or renal disease, if coexisting, may make the patient pale. The capillary vessels of the surface sometimes fill and become dark from congestion; or, as our author has seen, the extremities of the fingers and palms may present permanently an erythematic redness.

The physical signs of valvular lesions are carefully detailed by Professor Flint—chiefly obtained by auscultation, in which he is thoroughly an expert. They are divided by him under the heads of—1. Murmurs, new or adventitious sounds; and 2,—Abnormal heart sounds. We are glad that he has chosen to use the English word here with Forbes, and to discard the French *bruit*.

He divides murmurs with Latham into the endocardial and exocardial, including among the former those occurring in the large arteries near the heart. These are produced by the current of blood passing through the cavities or orifices of the organ, or those vessels, and resemble the sound heard in a bellows nozzle when air is driven out. The name of bellows murmurs, generally appropriate, fails to apply to some of the intracardiac murmurs. The exact localization of each of these sounds is ordinarily practicable, and hence their high diagnostic value.

It is true that there are inorganic murmurs which are not due to structural lesions; but these, with proper knowledge and care, can be distinguished. They arise from certain conditions which can and ought to be observed and appreciated; alterations in the composition of the blood, sudden diminution in the circulating mass, and functional disorder of the heart.

It is also true that valvular lesions involving great obstruction and regurgitation may exist without murmur; but such cases are rare and exceptional; nor is the degree of murmur proportioned always to the structural change which it manifests, diminishing and perhaps disappearing as the disease progresses. After all, however, our author concludes, as it seems to us with good reason, that although the absence of murmur is not absolute proof of the non-existence of valvular lesions, yet that the chance of error arising from this cause is so small that it need not affect our diagnosis.

He divides murmurs again into systolic and diastolic; the first accompanying, replacing or closely succeeding the first sound of the heart; the second closely preceding, accompanying or following or replacing the second sound. If the heart's action be so rapid as to occasion difficulty in deciding whether a murmur belongs to one or the other of those heads, the frequency may be reduced by sedatives, such as digitalis or veratrum viride. The distinction between the two systolic murmurs is readily made; the mitral regurgitant having its maximum of intensity at or near the apex of the heart; the aortic direct being best heard at or above the base of the organ in the second or third intercostal space near the sternum. The systolic murmur is generally soft, and the pitch moderately high.

The diastolic murmurs are feeble and less strongly marked. The mitral direct has its greatest intensity over the body or apex, and just precedes the first sound of the heart. The aortic regurgitant has its maximum intensity over the body of the organ near the sternum, and closely follows the second sound. It is always soft and its pitch usually low.

Inorganic or hæmic murmurs are uniformly systolic, and usually heard at the base of the heart. They are always soft and feeble—apt to be fluctuating and variable. We may accept the rule then that a murmur, if constant or persistent, intense, rough and mitral, or heard at the body or apex of the heart, is of organic origin. Concurrent bellows murmurs from the large arterial trunks, and the *bruit de diable*, which with Ward he regards as a venous hum, are usually associated with inorganic endocardial murmur of hæmic origin.

Our author calls attention to the modification of heart sounds connected with valvular lesions, as aiding us to localize them distinctly. Mitral lesions impair the mitral portion of the valvular element of the first sound. To isolate this sound, the stethoscope is to be placed without the left nipple sufficiently to eliminate the element of impulsion. They also lead to diminished intensity of the aortic second sound, and increased intensity, both of the tricuspid sound and of the pulmonic second sound. Aortic lesions impair the aortic second sound and even extinguish it.

Well-marked purring tremor, *fremissement cataire*, denotes valvular lesion, chiefly of the mitral orifice permitting regurgitation, and associated with hypertrophy of the left ventricle.

Of the prognosis in cases of valvular lesions, our author speaks with his usual caution. They are not necessarily fatal—nay, they often produce little or no inconvenience. He gives instances, one of them truly wonderful, of this tolerance. We are familiarly acquainted with an example of similar indifference in a youth, who, with most marked signs of organic disease, takes every form of exercise and enjoys all sorts of active recreation. All cases however of such lesion must be regarded gravely and with watchful supervision.

In the management of this class of patients we must recollect that the organic changes existing are irremediable, and that the measures usually resorted to by the unreflecting practitioner with a view to the removal of morbid deposit—such as alteratives, depletants, and counter-irritation, are useless, and worse. These anatomical changes are, however, progressive, and we must endeavor to retard their increase by those indirect methods which go to prevent overstraining of the valves, and renewal of the endocardial inflammation. Excessive muscular exercise, mental emotion, and stimulants excite unduly and overtask the heart, and must be avoided. Exposure to alternations of weather must be carefully guarded against as likely to give rise to rheumatism, the source of most of these derangements.

We must, however, assiduously obviate the tendency to weakness and dilatation of the heart, and preserve as fully as possible the tone of the system, as advised in treating of enlargements of the heart.

Occasional and secondary effects of valvular lesions, depending as they do chiefly on congestion, must be met by revulsive and palliative measures. Constipation is to be corrected. Dropsical effusion will require the use of diuretics and hydragogue cathartics, the former being preferred, if the kidneys readily respond to them.

Our author advises a certain reticency as to the communications to be made to the patient concerning his condition. Where the existence of lesions cannot be denied, a candid statement should be made, accompanied with such explanations as will divest the fact of greater importance than really belongs to it.

To his friends a cautious prognosis should be given. The progress of these cases is variable, and there is obvious liability to accident and incidental affections which may prove fatal unexpectedly; but patients often live long under very serious lesions.

We will pass over the fifth chapter on congenital malformations, misplacements, and defects of the heart and cyanosis, as containing no views either new or peculiar to our author. He offers a clear compendium of the collated facts and received opinions, referring to Peacock and Moreton Stillé, principally and frankly.

Next we have some ingenious reasonings on the subject of heart-clots and coagula, polypi so called, of ante-mortem and post-mortem formation. Our author does not doubt that coagulation takes place before death in certain cases, and suddenly arrests the circulation. This accident is supposed by our highest obstetric authority, Professor Meigs, to account for the quasi syncopic deaths occurring in certain puerperal cases attended with large hemorrhage. But Dr. Flint declares that such instances can be only conjectured, not positively known, as the clots found in the cavities of the heart do not differ from those formed by coagulation in the dead body.

Coagula formed before death are known by their density, the absence of red globules, their intertwining with the tendinous chords and fleshy columns, adhesion to the pericardium, and certain changes impressed upon the structure externally by the blood currents and internally by molecular disintegration. Professor Flint regards the fibrin of the blood as incapable of organization, and therefore is led to explain the instances in which these fibrinous masses seem grafted into the heart, by the supposition that the coagulated fibrin is deposited on an organized exudation of morbid growth. We do not consider the question, as to the character and capabilities of fibrin to be as yet absolutely settled; and if Virchow is right as to the unequivocal dependence of cell upon pre-existing cell, of ovum upon pre-existing ovum, we can as readily comprehend the growth of fibrinous strips, or masses, as of any other morbid products. Perhaps it is correct to say that we do not yet comprehend the point of departure in any morbid growth.

These fibrinous coagula are specially liable to be deposited during the progress of certain affections characterized by the presence of an undue proportion of this element in the blood, as in pneumonia, in epidemic cholera, and in endocarditis. An extraordinary case is described, in which the obstruction of the right auriculo-ventricular orifice was complete; the curtains of the tricuspid valve being literally tied firmly together by a dense colorless fibrin, intertwined with the tendinous chords and papillary muscles, and prolonged into the right auricle. The subject was found dead in his bed; but the heart was not enlarged, the valves were sound, and there were, except as described, no other morbid appearances. Neither the symptoms nor the physical signs are distinctive or diagnostic of these conditions. If we could distinguish them, the prognosis would be in the highest degree unfavorable, and all suggested treatment must be nugatory. Yet our author concludes, perhaps with a pardonable inconsistency, as it involves the only faint gleam of hope for our patients, that it is not improbable that certain remedies may favor the solubility of fibrin; and that as the researches of Richardson have led us to look upon its solution in the blood as owing to the presence there of ammonia, it would seem to be a reasonable inference that ammoniacal remedies may be efficient prophylactics.

Polypi are, though rarely, found in the heart, but are not to be confounded with heart-clots or fibrinous coagula. They are true pedunculated tumors, of fleshy or fungous aspect, and are formed slowly, their effects being developed gradually and imperceptibly. Neither the symptoms nor physical signs are at all distinctive.

A very interesting section follows, upon Angina pectoris. After a well-drawn description of the paroxysmal attacks of severe suffering, known under this appellation, Prof. Flint discusses with great ingenuity its character and relations. He affirms it to present a strongly-marked individuality. It is neuropathic; not connected essentially with any special organic change, or any of the known varieties of morbid anatomical determination. Yet he affirms it to be symptomatic of cardiac disease, and incidental exclusively to organic affections of the heart.

He distinguishes it from cardiac asthma, and maintains that dyspnœa proper does not belong to its history; the patient refraining from the respiratory movements voluntarily as he does from all other motion for fear of pain. Nor does he think it possible that spasm of the cardiac fibres should continue so long, consistently with life, as the paroxysm is known to endure. He views it therefore as a cardiac neuralgia, with which spasm and dyspnœa may be combined, and which is dependent upon unascertained cardiac lesions, but which is not in its nature connected with any one of them. He recites a case in which even the prehension of solid food determined an attack; the same miserable patient hardly daring to sleep, because even then he was liable to these paroxysms.

It is to be diagnosticated from pseudo-angina in the first place by a careful observation of its own characteristic symptoms, familiar to every reader, and therefore not repeated here; and in addition, by observing the usual connection of the neuralgic pains which simulate its paroxysm with the other marks of associated diseases, hysteria, dyspepsia, gout, anæmia, etc. There is less intense anguish, less immediate expectation of death; the attacks are not so abrupt, and continue longer; nor are the evidences of cardiac disorder so manifestly present.

In the treatment of angina pectoris immediate relief must be sought from opiates and diffusible stimulants. From the earliest discovery of chloroform we have been in the habit of employing it freely and with more benefit than any other or all other remedies put together, and Professor Flint mentions the marked relief produced from it in his severest case.

But it is evident that these, however efficient, are only palliative agents; we are yet unable to point out any truly preventive, or curative course of management. Happy he who shall make such useful discovery here. At present all we can do is to avoid as far as practicable all exciting causes which bring on the dreaded paroxysm; address ourselves to the diminution of the influence of coexisting cardiac or other disorder, and endeavor to keep up the tone of the constitution.

Next we have some curious remarks upon the enlargement of the thyroid body and prominence of the eyes, associated occasionally with cardiac affections. To the list of such cases, our author makes three additions; and we have more than once examined a similar instance in the wife of a brother physician, in which the gland is protuberant and the eyes project, with an obliquity of the axis of one of them.

The pathological character of these affections is as yet undetermined, and the nature of their connection with each other and with the morbid changes in the condition of the great central organ of circulation not understood. The treatment of them must be directed by a due attention to the coexisting morbid contingencies, of which anæmia, and in females, who constitute the great majority of subjects, disorders of the menstrual functions are the chief.

Reduplication of heart sounds constitutes the next topic of discussion. Its limited pathological import and want of diagnostic signification are fairly stated, while the mechanism is reasoned out with extreme plausibility from minute observation. The interest belonging to this subject, says our author, if the antithesis be allowable, is rather scientific than practical. He is not satisfied with the explanation of the phenomena on the supposition of mere non-synchronism of the contractions of the ventricles, although he admits that this want of unison is the most plausible hypothesis. The most remarkable example is one given by him, in which there existed, for seventeen days, four heart sounds for each pulse at the wrist; but the carotid was precisely twice as frequent as the radial pulse.

The patient, a sailor, aged twenty-seven, labored under a cough, with dyspnœa and some œdema of the face and lower limbs. He recovered after two months' residence in hospital, and died five years afterwards with some affection foreign to the heart. His body being examined, the organ was found moderately enlarged, without valvular lesion. During the illness above mentioned, the apex beat was not appreciable, though a feeble, short bellows murmur was heard at the apex; the heart being evidently enlarged. This murmur had disappeared, and the pulse and heart sounds were in normal ratio when he left the hospital.

The inflammatory affections of the heart are divided by Professor Flint, as is usual, into pericarditis, endocarditis, and carditis proper or myocarditis; the latter implying inflammation of the muscular substance, the others of the serous membranes investing and lining the organ.

Less frequent than endocarditis, pericarditis is a more serious and dangerous affection. It is not often idiopathic or insulated; and occurs both as acute and chronic. It is rarely fatal at an early stage. When this has happened, the membrane has been found reddened, mottled, and injected. It is opake, abnormally dry, or glutinous with the presence of lymph, which is soon deposited in a thin layer spread over the surface; this exudation going on, a serous liquid accumulates within the sac. If the patient do not sink at once, this liquid is gradually resorbed, and, the diseased surfaces of the sac falling into apposition, adhesion takes place.

This succession of events suffices to divide the attack into three stages: the first extending from the access to the point of time at which serous accumulation is determinable; the second embracing the period during which the amount of liquid is appreciable; and the third comprising the duration of the disease after its resorption.

In the majority of cases of fatal pericarditis, death occurs during the period of effusion. This is more or less prompt and rapid, having been distinguished within twenty-four or thirty-six hours, but not generally until three or four days have elapsed. In some cases from four to six ounces of fluid may be determinable; but the distention is sometimes enormous, as in a case reported by Swett, who found eight pounds within the sac. This great accumulation belongs, however, rather to the chronic than the acute form, in which it rarely exceeds two or three pints.

The adhesions which take place in more protracted attacks are either mechanical or by means of organization. Layers of lymph, simply agglutinating the sac to the heart with a certain force, never take on organization, and are called properly false membranes. But sometimes the intervening deposit of lymph is less abundant and dense, newly organized structures are formed, and adhesion takes place by a vital union, which may be so complete as totally to obliterate the pericardial sac. Our author met with a case in which adhesion had occurred over the left half of the organ, and a large amount of fluid was contained in the right half of the sac. The strength of these adhesions is the test of their duration or age.

The pathological relations of acute pericarditis are extensive and important. It is seldom primary or idiopathic. Its most frequent connection is with rheumatism: the ratio, according to Fuller, being about one in six cases. Our author gives the proportion of six in nineteen, recorded by himself. It most frequently manifests itself in early life and in first attacks of rheumatism, and more in females than in males. He met with one instance in which it preceded, and another in which it occurred coincidently with the affection of the joints. The nature of the relation is not clear. It is not a metastasis; each being the local expression of a constitutional affection, probably involving undefined blood changes.

With Bright's disease, also, pericarditis has an ascertained relation, somewhat better understood. The intermediate morbid condition determining the serous inflammation is supposed to be uræmia, the urea in the blood or the results of its decomposition acting with poisonous efficiency.

The coincidence, frequently observed, of pericarditis with pleuritis, or pleuro-pneumonia, our author regards as merely incidental, and dependent equally upon some common causative contingency. They are not always continuous, the pleuritis being sometimes on the right side.

Pyæmia frequently coexists with pericarditis in fatal cases. The eruptive and continued fevers also occur in the same complication, though rarely. Our author met with an instance of the development of pericarditis in scarlatina, being preceded by albuminuria and general dropsy. It has been met with in connection with scurvy, purpura, cyanosis, erysipelas, influenza, and tuberculosis of the lungs. Yet Professor Flint thinks that neither the tuberculous nor the carcinomatous cachexia gives rise to inflammation of the pericardium, except in the very few instances in which tubercle is deposited upon this membrane. Endocarditis coexists in a very large proportion of cases of pericarditis; almost without exception in rheumatic subjects.

The symptoms of pericarditis, as they affect the heart itself, are, pain in that region, palpitation, and tenderness on pressure. The general circulation is modified by the series of changes which take place; at first the pulse is strong, quick, vibratory, frequent, and irregular; when the movements of the central organ are restrained by the pressure of effusion in the sac, it is notably small and weak, with marked disturbance of its rhythm. It varies greatly at times, without obvious cause. Lividity of the lips and face may be due merely to the oppression of the heart's action. Œdema, in general, implies coexisting renal disease, or organic cardiac lesion. The respiration is variously affected; there may be notable dilatation of the alæ nasi; cough is commonly but not uniformly present; dyspnœa may be wanting, though a usual concomitant, even in the degree of orthopnœa. The digestive system is sometimes so greatly though incidentally disturbed that the vehement and persisting vomiting has occasioned the attack to be mistaken for gastritis. Dysphagia is also of occasional occurrence.

There is frequently a marked expression of anxious apprehension in the countenance, and late in severe cases the risus sardonicus is seen. The position of the patient is usually on his back, inclining to the right, and he is very reluctant to move. Fatal syncope is sometimes suddenly induced by rising.

Professor Flint draws attention to the disorders of the nervous system associated with pericarditis. Cerebral symptoms are not seldom developed which are not only highly important in themselves, but especially as marking the symptoms of cardiac disease. Inflammation of the meninges of the brain, mania, dementia, coma, epilepsy, tetanus, and chorea, have been simulated in cases of pericarditis; the latter disease being generally overlooked before death, and examination *post mortem* revealing no appreciable lesions of the brain or spinal cord adequate to explain the phenomena during life. Our author recites three cases of curious interest: two of them fatal; the third recovering from an attack of fearful violence. Among the forms of delirium observed here, a fixed delusion of having committed some crime appears to be a distinguishing feature. Of the nature of the pathological connection assumed to exist between these morbid affections, we know but little. They concur too often to be mere coincidences. The nervous symptoms are to be regarded as functional, not leaving any palpable traces in the brain or spinal cord. They are probably the results of a common causative influence.

Of the physical signs of acute pericarditis, we have a summary which it is difficult to abbreviate without losing something of its admirable clearness.

Percussion shows an enlarged area of præcordial dullness, extending more vertically than transversely, the dullness and sense of resistance being greater than in enlargement of the heart. These signs indicate abundant effusion within the sac. We note the prompt and rapid increase of the area of dullness, within a few hours or days, as showing progress of the disease; and diminution of that area proportionally, in the progress toward convalescence.

Auscultation detects a friction sound, developed soon after the commencement of the attack, depending generally on the effusion of lymph; rarely wanting in the first stage; frequently but not uniformly disappearing during the second, or period of effusion; often returning after the absorption of the fluid, and persisting even when adhesions have taken place. The heart sounds are at first intensified and then become weaker; the element of impulsion in the first sound impaired or lost, and the valvular left alone; the sounds apparently distant, and more so in some positions than others. The respiratory murmur and vocal resonance are lost over the enlarged area of præcordial dullness determined by percussion.

Palpation discovers the cardiac impulse at first unduly violent, forcible, sometimes tumultuous, extending abnormally. After effusion, the apex beat is raised and carried to the left, and suppressed if the effusion be large. There is vibration of the thoracic walls in the præcordia, constituting tactile friction-fremitus. The apex beat is in some cases notably retarded by a certain amount of effusion. Resorption allows the return of the apex beat to its normal position and conditions.

On inspection and mensuration we shall find distinct prominence of the præcordia, with restraint of the respiratory movements of the left side from pain or large effusion. Depression of the præcordial region follows in some cases the resorption of the effused fluid.

The diagnosis of pericarditis can only be effected by careful physical explorations. These will suffice to distinguish it from all morbid affections, except hydro-pericardium without inflammation. And even here, though the same marks of distention be present, we separate them by noting the friction sound seldom wanting, and collating the attendant symptoms—pain, tenderness, febrile movement. In hydropericardium there is no friction sound; œdema or anasarca, ascites and hydrothorax are present.

The prognosis is laid down with habitual caution. The attack of pericarditis is never trivial, often formidable. Its mortality, however, is generally due not so much to the disease itself as to associated circumstances. It rarely proves fatal as connected with rheumatism; its combination with Bright's disease is almost invariably so.

Its ordinary duration is from one to two weeks; it has terminated unfavorably in twenty-seven hours. If protracted, it runs into the chronic form. The termination of fortunate cases is apt to be attended with adhesions.

The mode of death, in acute attacks, is by syncope or gradual asthenia; the arrest of the circulation, owing to paralysis of the heart, oppressed by the effusion and influenced by the proximity of the inflamed membrane to the muscular fibres of the organ. Concomitant affections, however, may determine the fatal result, as by coma, apoplectic or uræmic.

In the treatment of acute pericarditis, our author permits the reticent employment of the lancet, though rarely; mercurialization he refuses to condemn utterly, discriminating in its favor in idiopathic and rheumatic cases, and denouncing it as inappropriate in anæmia, and where Bright's disease exists. Sedatives are rarely indicated, and only admissible before effusion. Revulsive measures, such as sinapisms, fomentations, are useful in the early stage. Vesication, at a late stage, he thinks promotes absorption. Opium should be given in doses sufficient to tranquilize and to relieve pain. Eliminatives are appropriate, under due restrictions, where the disease is dependent on rheumatism or uræmia. The most prominent of these are the various diuretics, the alkalies, and colchicum. Cathartics and sudorifics are of doubtful utility.

In the progress of the attack a nutritious diet must be given, and diffusible stimulants as freely as they can be well borne. Alcoholic fluids, either wine or spirits, are the best and most efficient of these. Animal essences or tender animal solids must constitute the diet generally.

To promote the complete disappearance of inflammation, and thus expedite the process of resorption of the liquid effusion, we must persist in counter-irritation, by perpetuated blisters, and pustulation with croton oil. We should take care not to press these measures so far as to produce constitutional disturbance or exhaust the patient.

In subacute or chronic pericarditis, the cases present very different anatomical conditions. These our author arranges into two classes, viz.: 1st. Absence of liquid effusion, the pericardial surfaces being agglutinated by interposed layers of lymph, of variable thickness, with imperfect organized attachment, or none. 2d. More or less distention of the sac with turbid serum, or puruloid or truly purulent liquid.

When without effusion, these cases are obscure. Acute pain is rarely present; nay, there may be entire absence of all subjective symptoms. An uneasiness and constriction are apt to be referred to the præcordia, and palpitation with dyspnœa follows muscular exertion. Lymph is abundantly deposited in layers, which may be peeled off the heart's surface. These layers are dense, and of quasi membranous structure, but are not organized; they adhere to the visceral and parietal surfaces of the sac, perhaps firmly, but by mere mechanical agglutination. Friction sounds are sometimes heard. Cases of the second class, with effusion, are more readily known. The amount collected is sometimes enormous, and the symptoms produced such as have already been recited.

To what has been said on the subject of treatment above, may be added the suggestion of the external application of iodine in the first class of cases, and perhaps its internal use also as sorbifacient or promotive of the removal of the morbid deposits. When the pericardial sac is largely dilated, it is proposed to empty it by paracentesis. Aran has had the boldness to inject, after drawing off the liquid, a solution of iodine, and it is said with apparent benefit.

A brief section on pneumo-pericardium and pneumo-pericarditis contains the history of some examples of these rare affections. Air or gas may gain access within the sac by fistulous communication with the stomach, intestines or lungs; or through perforating wounds; or as generated in the cavity. The physical signs which attend these uncommon accidents are—metallic tinkling, a splashing or gurgling sound produced by the heart's action, tympanitic resonance, and the bruit de pot fêlé on percussion.

Pericardial adhesions are very frequent as consequent on inflammation of the sac—nay, it is doubtful whether recovery can take place without them. They may be general or partial, the former occurring most often. They are found associated in a certain proportion of cases with valvular and other cardiac lesions; and also, not seldom, when there has been no suspicion of cardiac disease. In the discussions concerning their influence and importance, so warmly entertained, our author is led to agree with those who consider their evil tendency to be far less than was supposed by Hope and the older pathologists. He does not deny that they may contribute to enlargement of the heart in a certain proportion of cases; nor that in rare instances they may tend also to an opposite result; but declares that they exist not unfrequently, for a long period, without being followed by any appreciable morbid condition, or the production of any organic change or functional disturbance.

To the question, how their existence is to be ascertained during life, he answers, that the diagnosis cannot be always made with positiveness, but that the physical signs may in a certain proportion of cases be relied on with much confidence; and points them out as follows: the area of præcordial dullness on percussion being fixed and unaltered by changes of posture, and unaffected by inspiration, however deep; the limits of the respiratory murmur not affected by a deep inspiration; the apex beat affected slightly or not at all by changes of posture or forced respiration; retraction of one or more intercostal spaces, together with the ribs, in some cases, and depression of the epigastrium synchronously with the heart's systole. The inference drawn from these signs will be confirmed, if there be contraction of the chest, limited to the præcordia, and if we learn that the patient has formerly suffered from pericarditis or from acute rheumatism.

Endocarditis, recently recognized with nosological distinctness, now known as not infrequent, is next fully treated of. Its coexistence with acute rheumatism, in a large proportion of cases, is considered by our author to be one of the most important developments of modern medicine. Its remote effects in the production of valvular lesions, already discussed, invest it with great interest. We rarely meet with a case proving fatal in its early stages, and therefore know less of the morbid appearances presented during its progress than of its ultimate results. It is chiefly seated in the left side of the heart, and is generally limited to these portions of the membrane covering the valves and lining the orifices. This is attributed to the anatomical fact, that it is here underlaid by fibrous tissue, while elsewhere it is in proximity to the muscular substances.

Professor Flint lays no stress upon the redness of the membrane as an evidence of inflammation, but dwells rather on the structural changes of condition. These are roughness, villosity, opacity, swelling and softening. Atheromatous deposit and hypertrophy of the endocardium, as arising from morbid processes, not inflammatory, and ancient inflammation, are to be distinguished from the above. The membrane is in direct contact with the blood, and not being a shut sac, like other serous tissues, presents two sources of morbid deposit or product. Besides the ordinary exudation of lymph occurring here, as in all serous inflammations, the fibrin of the blood is liable to be deposited by coagulation, to attach itself to certain points, and again to be detached and washed away into the circulating current. The two deposits become mingled; the roughness from the exudation of lymph or other alteration of the membrane attracts, as a foreign substance, and retains the fibrin thus precipitated or coagulated. The exudation of lymph is more unequivocal evidence of inflammation than the deposit of fibrin. Other morbid changes still are met with, as ulceration, erosion, perforation and laceration of the valves, and occasionally gangrene.

Endocarditis is rarely idiopathic, and seldom occurs, indeed, in any other connection than as associated with acute articular rheumatism. Our author announces the opinion—we cannot help hoping, somewhat more unfavorable than the general experience of our country will warrant—that in any case of that affection the chances are about equal that the endocardial membrane will become involved. Authorities differ as to the relation between endocarditis and pericarditis. Our author agrees with Hope, that the former is more apt to occur alone of the two, but affirms their not infrequent combination. Like pericarditis, it is liable to be developed in connection with Bright's disease, and this uræmic form is much more likely to prove fatal than the rheumatic. Pulmonary inflammation exerts some influence, too, though feeble, in determining the occurrence of endocarditis.

The formation of coagula in the cavities of the heart is among the immediate pathological effects of inflammation there. These heart-clots and the emboli, consisting of detached masses of lymph or fibrin, or both, propelled into the arteries with the blood current, and carried in the smaller vessels, thus producing obstruction, are supposed to give rise to apoplectic and epileptiform seizures, paralyses, etc. The liability to their occurrence should be kept in mind; but it must be rare, in view of the fact that endocarditis is fatal in so very small a proportion of instances.

The diagnosis of endocarditis rests on physical evidence, and this consists in the development of endocardial murmur, in connection with symptoms fixing its significance; as, chiefly, the presence of acute rheumatism. With commendable frankness, Professor Flint acknowledges that he is unacquainted with any method of recognizing idiopathic endocarditis, which, however rare, occurs in children, as there is reason to believe, without being detected.

In the treatment of this affection, our objects are the abatement of the intensity of the inflammation, abridging its duration, limiting the exudation of lymph and precipitation of fibrin, and effecting the removal of solid deposits. The measures adapted to attain these purposes are those indicated in the treatment of pericarditis, and must be employed with the same cautions and limitations; blood-letting and other antiphlogistic remedies, mercurials, opium, sedatives, eliminatives, and counter-irritants. We are warned against the error, not seldom committed, of persisting in the application of these means of combating inflammation, long after it has ceased, although it is not always easy to determine when that period has arrived.

Myocarditis, inflammation of the muscular substance of the heart, is so seldom met with, that very few cases are on record. Its presence cannot be brought within the reach of diagnosis, nor, if we could recognize it, would there be any peculiarity of management suggested.

The ninth chapter is devoted to functional disorder of the heart, the several forms of which are briefly but graphically described. It is usually paroxysmal; the movements of the organ being irregular and tumultuous, sometimes intermittent. There is great despondency and fear of impending death. The paroxysms differ in severity, and vary in duration from a few seconds to several hours. The immediate exciting causes are muscular exertion, mental emotion or passion, ingestion of food or of stimulants. We meet with rare cases of merely persistent inordinate action, in which there is abnormal rapidity and force without marked irregularity of any kind. Again, there is great feebleness of contraction, with tendency to syncope—tremulousness or interruption.

These phenomena present themselves as connected with plethora, anæmia, nervous derangement, dyspepsia, and the gouty diathesis. They may be noticed during rapid growth and in convalescence from fevers.

There is nothing specific in the treatment, which must be directed to the contingencies upon which these disturbances are dependent.

A careful diagnosis is required, and must be obtained by physical exploration, collated with minute recognition of collateral details. This diagnosis involves more than the mere question of the presence of organic disease, which may perhaps be readily answered negatively or affirmatively. But we must not lose sight of the fact that functional disorder and organic disease may be associated, and yet the former not be dependent on the latter. We are led to this favorable inference by the following considerations. The disturbance is disproportioned in degree to the ascertained anatomical changes; it is paroxysmal, with intervals of complete or great relief; it is more likely to happen in the young and in females; attacks come on at night, or upon the application of obvious exciting causes; there is not likely to be any lividity, nor œdema, nor general dropsy.

Under these circumstances we may venture reasonably upon a favorable prognosis, for experience has proved the falsity of the supposition formerly entertained, that functional disorder, if protracted, eventuates in organic disease. There is no ground for the belief. For the palliation or arrest of the paroxysms above described, opiates are advised, antispasmodics, carminatives, revulsives, and, if the patient be feeble, stimulants.

The book concludes with a notice of diseases of the aorta, and thoracic aneurisms.

Acute aortitis cannot be diagnosticated; nor do symptoms or physical signs furnish the means of recognizing, during life, the subacute or chronic form of inflammation of the great artery. But we meet in post-mortem examinations with many anatomical changes, ascribed to this latter as a cause. Morbid deposits are often found, chiefly but not exclusively in aged males, situated upon the internal surface of the vessel, forming patches of a white, dense substance, membraniform, or of cartilaginous firmness. The union, though close, is not of organized attachment.

Still more common is the deposit known as atheroma. It is more or less hard or soft, even semi-liquid sometimes, and forms patches of various dimension. The microscope shows it to contain oil globules, with crystals of cholesterine. It is a variety of fatty degeneration, and is often associated with fatty heart. It becomes the seat of calcareous deposit, and thus the patches are transformed into plates or masses resembling bone; true ossification, however, does not occur.

Of these changes, a not infrequent result is dilatation of the ascending aorta. This may be known, according to Bellingham, by the characteristic signs of visible pulsation of the large arteries of the neck and upper extremities, with a pulse, at the wrist, jerking or receding. Our author speaks with less confidence of the diagnosis.

Among the various forms of thoracic aneurisms, scientifically known and described, Prof. Flint treats only of those which are distinguished as sacculated. To be recognized, they must have attained considerable bulk, and their situation chiefly determines their symptoms and signs. They are seated ofttimes in the ascending aorta; next in the arch; and less frequently in the descending aorta. They occur at the sinuses, behind the semilunar valves; rupture takes place readily, on account of the absence of the cellular coat of the vessel at this spot, and death is sudden. Diagnosis here is impossible. Of two cases happening under the observation of our author, one had been, up to the time of death, in apparently perfect health; the other had recovered from an attack of pleurisy. No symptoms in either had indicated disease of the heart or large vessels.

Thoracic aneurism generally shows itself by the bulging of an external tumor—conical, smooth, pulsatory. Pulsation is often felt before any protrusion takes place; it is sometimes, though rarely, wanting. There is often, but not always, tactile fremitus. The bellows murmur is heard usually, upon auscultation, over the tumor, but is of less value as a sign than has been generally supposed. It may be single or double. There is wheezing or stridulous breathing, from pressure on the trachea; a bronchus may be so compressed as to arrest respiration in an entire lung. Dysphagia may arise from obstruction of the œsophagus; aphonia, or hoarseness, from interference with the larynx. Paraplegia follows erosion of the vertebræ and pressure on the spinal cord. Hemiplegia has been known to attend, whether as a mere coincidence or as the remote effect of disturbance of the cerebral circulation.

The mode of death is usually by rupture or opening of the aneurismal sac, and prompt hemorrhage. But, in some instances, pressure on important parts—the trachea, bronchi, lungs, œsophagus, spine, vena cava, and pulmonary artery—occasions an earlier termination. This may also depend upon emboli, detached from within the sac.

Recovery from thoracic aneurism is possible, but hardly possible.

When a spontaneous cure has taken place, the sac, filled with fibrin, and remaining attached to the artery, presents the appearance of an extraneous tumor, and undergoes obvious reduction in size, in progress of time, from the contraction of the fibrin.

The treatment of these cases must depend on circumstances. A plethoric patient must be cautiously depleted; in anæmia, we must prescribe a nutritious animal diet, with iron, and perhaps stimulants. The use of liquids is to be limited. Everything which induces cardiac excitement must be avoided, and the patient encouraged and consoled with hope of slow progress, or the possible arrest of the disease. Sedatives may be employed to regulate the heart's action, such as digitalis, opium, prussic acid, aconite, and hyoscyamus. Acetate of lead, and tannin or gallic acid, have been recommended as tending to promote coagulability and coagulation of the blood fibrin. In the mean time, we should not neglect the measures proper to palliate the irremovable evils associated with the presence of the tumor, and relieve, as much as lies in our power, the pain suffered by the miserable patient—the dyspnœa, cough, dysphagia and cerebral obstruction, and congestion.

We have thus endeavored to present our readers with a fair analysis of this remarkable work. Preferring to employ the very words of the distinguished author, wherever it was possible, we have essayed to condense into the briefest space a general view of his observations and suggestions, and to direct the attention of our brethren to the abounding stores of valuable matter here collected and arranged for their use and instruction. No medical library will hereafter be considered complete without this volume; and we trust it will promptly find its way into the hands of every American student and physician.

